# Specific *in situ* immuno-imaging of pulmonary-resident memory lymphocytes in human lungs

**DOI:** 10.3389/fimmu.2023.1100161

**Published:** 2023-02-10

**Authors:** Duncan C. Humphries, Richard A. O’Connor, Hazel L. Stewart, Tom M. Quinn, Erin E. Gaughan, Beth Mills, Gareth O.S. Williams, James M. Stone, Keith Finlayson, Martine Chabaud-Riou, Florence Boudet, Kevin Dhaliwal, Vincent Pavot

**Affiliations:** ^1^ Translational Healthcare Technologies Group, Centre for Inflammation Research, Queen’s Medical Research Institute, The University of Edinburgh, Edinburgh, United Kingdom; ^2^ Research & Development, Sanofi, Marcy L’Etoile, France; ^3^ Centre for Photonic and Physics, Bath University, Bath, United Kingdom

**Keywords:** resident memory T cells, resident memory B cells, lung, optical endomicroscopy, fluorescence lifetime imaging

## Abstract

**Introduction:**

Pulmonary-resident memory T cells (T_RM_) and B cells (B_RM_) orchestrate protective immunity to reinfection with respiratory pathogens. Developing methods for the *in situ* detection of these populations would benefit both research and clinical settings.

**Methods:**

To address this need, we developed a novel *in situ* immunolabelling approach combined with clinic-ready fibre-based optical endomicroscopy (OEM) to detect canonical markers of lymphocyte tissue residency *in situ* in human lungs undergoing *ex vivo* lung ventilation (EVLV).

**Results:**

Initially, cells from human lung digests (confirmed to contain T_RM_/B_RM_ populations using flow cytometry) were stained with CD69 and CD103/CD20 fluorescent antibodies and imaged *in vitro* using KronoScan, demonstrating it’s ability to detect antibody labelled cells. We next instilled these pre-labelled cells into human lungs undergoing EVLV and confirmed they could still be visualised using both fluorescence intensity and lifetime imaging against background lung architecture. Finally, we instilled fluorescent CD69 and CD103/CD20 antibodies directly into the lung and were able to detect T_RM_/B_RM_ following *in situ* labelling within seconds of direct *intra-alveolar* delivery of microdoses of fluorescently labelled antibodies.

**Discussion:**

*In situ*, no wash, immunolabelling with *intra-alveolar* OEM imaging is a novel methodology with the potential to expand the experimental utility of EVLV and pre-clinical models.

## Introduction

Human and non-human primate (NHP) studies have highlighted the protective role of lung-resident memory T cells (T_RM_) in controlling respiratory pathogens (1–3). Constituting the majority of T cells within the lung (4), T_RM_ have a unique phenotype that differentiates them from other memory T cell subsets and their sequestration within the lung offers an ideal location to respond to respiratory infections (5). Requiring only cognate antigen for their activation (6), T_RM_ are highly proliferative, producing polyfunctional progeny with superior effector function (7, 8). Activated CD4^+^ and CD8^+^ T_RM_ in the lungs of patients with *Mycobacterium tuberculosis* (MTB) limit intracellular MTB replication in macrophages (9), whilst increased numbers of CD8^+^ T_RM_ in BAL is associated with reduced symptoms and viral load following human respiratory syncytial virus (RSV) infection (1). In human influenza infection, CD4^+^ and CD8^+^ T_RM_ confer heterosubtypic protection (10), with CD8^+^ T_RM_ recognising universally conserved peptides expressed by influenza A, B and C viruses (11). Virus-specific pulmonary CD8^+^ T_RM_ also have shown “innate like properties” that amplify inflammation and enhance neutrophil recruitment following noncognate bacterial infection to aid clearance, as demonstrated in murine models (12). While most actions of T_RM_ benefit the host, following chronic exposure to allergens, T_RM_ can also be detrimental and contribute significantly to pathology in experimental allergic asthma (13, 14), indicating monitoring the frequency and phenotype of T_RM_ has relevance beyond respiratory infections.

Antigen-experienced lungs are also enriched with B cells expressing a resident memory phenotype (15). Murine parabiosis studies have demonstrated that pulmonary-resident memory B cells (B_RM_) require local antigen encounter and early CD40/CD40 ligand interactions with T cells for their formation and are phenotypically and functionally distinct from their systemic counterparts (5, 16). Murine B_RM_ play a protective role in response to both viral influenza (16–18) and bacterial pneumococcal pneumonia (15) infections, providing rapid antibody secreting cells (ASC) capable of producing a range of class switched, cross-reactive antibodies to confer heterosubtypic protection (15–17).

Vaccination strategies designed to induce pulmonary resident memory lymphocyte populations have been successfully demonstrated in murine models (19). In NHP, pulmonary mucosal delivery of MTB vaccine is associated with enhanced protection over standard intradermal immunization *via* the induction of lung T_RM_ (20). As T_RM_ and B_RM_ formation is dependent on pulmonary cognate antigen encounter (16, 21), it’s anticipated that pulmonary mucosal vaccination will also generate B_RM_. This can induce higher levels of local immunity than that generated by natural MTB infection (20). Strategies that establish antigen-specific T_RM_ and B_RM_ populations or enhance pre-existing populations have the potential to provide local long lasting heterosubtypic protection where it is most needed. Current methods for evaluating respiratory vaccine efficacy, however, rely mostly on peripheral blood sampling to reveal the level of humoral protection and circulating central memory T cell (T_CM_) and effector memory T cell (T_EM_) populations. However, as T_RM_ and B_RM_ do not circulate and limited blood biomarker surrogates exist, current methods for assessing local immunity are limited.

These studies highlight the pivotal role of T_RM_ and B_RM_ in orchestrating and directing protective responses to respiratory infections. The spatial and temporal characterisation of such pulmonary resident immune cells is thus of key importance, but most available methods require biopsy, tissue digestion and post-mortem studies (16, 22). In this study, we aimed to develop an *in situ* method for the direct detection of these cells in the human lung by exploiting recent advances in *in situ-*optical endomicroscopy, within the alveoli (23), which may provide a valuable tool for quantifying pulmonary resident memory populations following infection and vaccination.

The objective was to develop a technical platform for direct alveolar imaging of pulmonary T_RM_ and B_RM._ We aimed to develop an *in situ* immunolabelling approach with a clinic-ready fibre-based optical endomicroscopy (OEM) system that combined both intensity imaging and fluorescence lifetime imaging (FLIM) to ensure contrast and specificity was achieved over endogenous elastin fluorescence of the lung. With NHP providing an ideal model for translational studies, this project also aimed to establish a cross-reactive antibody panel capable of detecting T_RM_ and B_RM_ in both humans and NHP species.

FLIM is a fluorescence imaging technique where the contrast is based on the lifetime of individual fluorophores rather than their emission spectra. The fluorescence lifetime (FLT) is defined as the average time that a molecule remains in an excited state prior to returning to the ground state by emitting a photon. The FLT of the fluorophore, rather than its intensity, is used to create the image in FLIM. FLT depends on the local micro-environment of the fluorophore, thus precluding any erroneous measurements in fluorescence intensity due to change in brightness of the light source, background light intensity or limited photo-bleaching (24).

A clinic-ready fibre-based FLIM system (KronoScan), incorporating a time-resolved spectrometer and an achromatic advanced confocal laser scanning microscope system was used coupled to a fully biocompatible multifunctional endoscopic fibre (Panoptes) with an outer diameter of 1.8 mm, which comprises an imaging fibre bundle packaged alongside two capillary channels for delivery and extraction of fluids

T cells recovered from bronchoalveolar lavage and lung tissue digests from *ex vivo* whole human lungs showed characteristic features of T_RM_ with CD69^+^CD103^+^ CD8^+^ T_RM_ being the most abundant population in the BAL and higher than in the lung tissue. A cross reactive panel of antibodies detected T cells bearing T_RM_ markers in NHP lung, although at much lower levels than in human lung. Cells isolated from human lung digests co-expressing CD69 and CD103 could be visualised using fibre-based imaging *via* both fluorescence intensity and lifetime imaging when labelled with anti-CD69 and anti-CD103 antibodies, both *in vitro* and after instillation into human lungs. Using KronoScan with Panoptes (25), dual antibody labelled cells (expressing markers of tissue retention) were detected *in situ* within human lungs undergoing EVLV within seconds of direct *in situ* delivery.

Our study extends the methods available for live imaging of leukocytes at the host-environment interface of the lung and demonstrates the potential of specific immunolabelling for distal lung interrogation. The *ex vivo* human lung model, closely resembles the *in vivo* situation, enabling validation of imaging modalities and facilitates transfer to both research and clinical settings.

## Methods

### Tissue source

Human lungs from deceased subjects deemed non-suitable for transplantation were received from National Health Service Blood and Transplant (NHSBT), United Kingdom. Following explantation and perfusion, lungs were placed on ice and transported to the University of Edinburgh within 48 hours prior to ventilation/lavage. Whole NHP (cynomolgus macaque from Mauritius) lungs were obtained from Charles River Laboratories (Edinburgh, UK) and transported in Roswell Park Memorial Institute (RPMI) 1640 medium (11875-093, Gibco) prior to tissue digest. NHP lungs were non-perfused and obtained from healthy control animals not subjected to experimental manipulation.

### *Ex vivo* lung ventilation

Human lungs undergoing EVLV were placed on a Dräger Savina 300 ventilator following placement of an endotracheal tube and ventilated with a tidal volume of 7 mL/kg (patient body mass) at a respiratory rate of 12 breaths per minute (**Supplementary Figure 1A**). Ventilation was volume controlled, air driven, and positive end expiratory pressure (PEEP) was set at 5 cm H_2_O. The airway pressure limit setting on the ventilator was increased to the maximum setting to prevent under ventilation.

### Bronchoalveolar lavage

Bronchoalveolar lavage (BAL) was obtained from human lungs undergoing EVLV by instilling 100 mL phosphate-buffered saline (14190-094, PBS, Gibco) into the right middle lobe or selected lobes *via* a bronchoscope. Aspirated cells were passed through a 40 µm cell strainer (431750, Corning, USA) and counted using a NucleoCounter cell counter (Chemometec, Denmark). Cells were processed fresh or cryopreserved in liquid nitrogen using Recovery™ Cell Culture Freezing Medium (12648010, Thermo Fisher).

### Lung tissue digest

Human (EVLV-derived samples from the distal lung) and NHP lung tissues were dissected with a scalpel and finely cut with scissors prior to incubation in 37°C DMEM (21969-035, Gibco) containing 1 mg/mL Collagenase IV (C5138, Sigma-Aldrich) and 0.1 mg/mL DNASE I (DN25 – Sigma Aldrich) for 1 hour with constant agitation. The sample was strained through a 70 µm cell strainer (22-363-549, Thermo Fisher) and spun at 350 x g for 5 minutes at room temperature. Red blood cell (RBC) lysis was performed using RBC lysis buffer (320301, Biolegend) for 10 minutes at room temperature prior to addition of DMEM and spun for another 5 minutes at 350 x g. Cells were counted using a NucleoCounter and processed for direct flow cytometry analysis or cryopreserved.

### Peripheral blood mononuclear cells

Human peripheral blood mononuclear cells (PBMCs) were isolated from whole blood using Percoll gradients (26). PBMCs were then cryopreserved. Cryopreserved cynomolgus PBMCs were obtained from BioIVT (BioIVT, United Kingdom).

### Flow cytometric analysis of lung tissue and BAL

Fresh lung digest and BAL cells were placed in 5 mL flow cytometry tubes. For cryopreserved cells, cells were gently warmed in 37°C water, placed in DMEM containing 10% foetal calf serum (FCS) and washed at 350 x g for 10 minutes prior to transfer to flow tubes. Cells were stained with live/dead marker Zombie UV (423107, Biolegend) in PBS for 30 minutes in the dark at room temperature. Cells were washed with FACS buffer (PBS + 2% FCS) and incubated with Fc block (564220, BD Biosciences) for 10 minutes prior to cell surface antibody staining containing BD Brilliant Horizon™ Staining Buffer (563794, BD Biosciences) (for details of flow cytometry antibodies used, see **Supplementary Table 1**). Following a 25-minute incubation in the dark at 4°C, cells were washed at 350 x g for 5 minutes and resuspended in Fixation Buffer (420801, Biolegend) for 20 minutes at room temperature before spinning at 350 x g for 5 minutes and resuspending in FACS buffer. For intracellular Ki-67 staining, cells were washed following cell surface antibody staining and processed using the Foxp3/Transcription Factor Staining Buffer Set (00-5523-00, Thermo Fisher Scientific) according to the manufacturer’s instructions. Ki-67 antibodies were used at dilutions recommended by the manufacturer (**Supplementary Table 1**). Samples were assessed using a 5L LSR Fortessa flow cytometer (BD Biosciences). Compensation was calculated using UltraComp eBeads™ (01-222-42, Thermo Fisher Scientific). Data analysis was performed using FlowJo software, version 10.7.2 (BD).

### T_RM_/B_RM_ immunofluorescence staining

Human and NHP lung tissues were placed in optimal cutting temperature compound (OCT, KMA-0100-00A, CellPath, UK) and snap frozen in liquid nitrogen. 10 µm sections were cut on to Super Frost microscope slides (12312148, Thermo Fisher) using a cryostat. Sections were air dried overnight at room temperature and then fixed in -20°C acetone for 10 minutes. Sections were air dried for <20 minutes prior to x2 washes with PBS. Multiplex immunofluorescence was performed on lung sections using a fully automated Bond-Rx Multiplex IHC Stainer (Leica Biosystems) and OPAL Multiplex IHC Detection Kit and counterstained with DAPI (Akoya Biosciences) according to a previously published protocol (27). T_RM_ were stained with purified, cross-reactive CD4 (OKT4, Biolegend), CD8 (SK1, Biolegend), CD69 (FN50, Biolegend) and CD103 (2G5.1, Bio-Rad, UK) antibodies. B_RM_ were stained with purified, cross-reactive CD20 (2H7, Biolegend), CD27 (M-T271, Biolegend) and CD69 (FN50, Biolegend) antibodies. Human lung (n=5) and NHP lung (n=3) sections were stained simultaneously using the same protocol and antibody concentrations. For full details, see online supplement.

### Fluorescence intensity and lifetime imaging system (KronoScan) and fibre (Panoptes)

The clinic-ready fibre-based FLIM system (**Supplementary Figures 1B–D**) (KronoScan), incorporates a time-resolved spectrometer (with 32 lifetime channels) and achromatic advanced confocal laser scanning microscope system, using a supercontinuum laser (SuperK EVO, NKT Photonics, Denmark) filtered to excitation band 490 nm and 590 nm. 50 frame fluorescence intensity and lifetime video sequences were simultaneously recorded using 128 x 128-pixel images (350 x 350 µm) matching fibre bundle characteristics. Spectral emission ranges of 498-565 nm and 620-764 nm were selected for analysis. Lifetime decays were fitted using rapid lifetime determination method (28), which assumes a single exponential decay, enabling high-processing speeds and real-time imaging at 3 frames per second (20 µs exposure time).

Panoptes (25) is a fully biocompatible multi-functional endoscopic device, with an outer diameter of 1.6 mm. It comprises an imaging fibre bundle packaged alongside two capillary channels for delivery and extraction of fluids. The low-index contrast imaging fibre (0.3 numerical aperture (NA)) is derived from multi-mode telecommunications preforms (OM1 PCVD rods). It is fabricated by multi-stacking arrays of different sized cores to optimise pixel density and suppress core-to-core crosstalk (29). This high-resolution OEM & FLIM imaging fibre, which comprises 8100 cores, with 450 µm diagonal field of view, is deliverable *via* existing bronchoscopic and transthoracic platforms.

### Fluorescence intensity and lifetime T_RM_/B_RM_ fibre imaging *in vitro*

5x10^6^ human lung digest cells or PBMCs were stained with 15 µL FITC-conjugated CD69 (FN50, Biolegend) and PE-Dazzle CD103 (BerACT8, Biolegend) to identify T_RM_, or 15 µL FITC-conjugated CD69 and PE-Dazzle-labelled CD20 (2H7, Biolegend) to identify B_RM_, for 20 minutes at 4°C in FACS buffer. Cells were spun at 350 x g for 5 minutes, resuspended in 100 µL PBS and transferred to a black 96-well plate. Fluorescence intensity and lifetime imaging was performed using OEM KronoScan Imaging system and Panoptes Multifunctional fibre. Videos were saved retrospectively when events were detected and analysed using the KronoScan Player (Firefinch Software Development, UK).

### *In situ* detection of T_RM_/B_RM_

**Figure 1:**
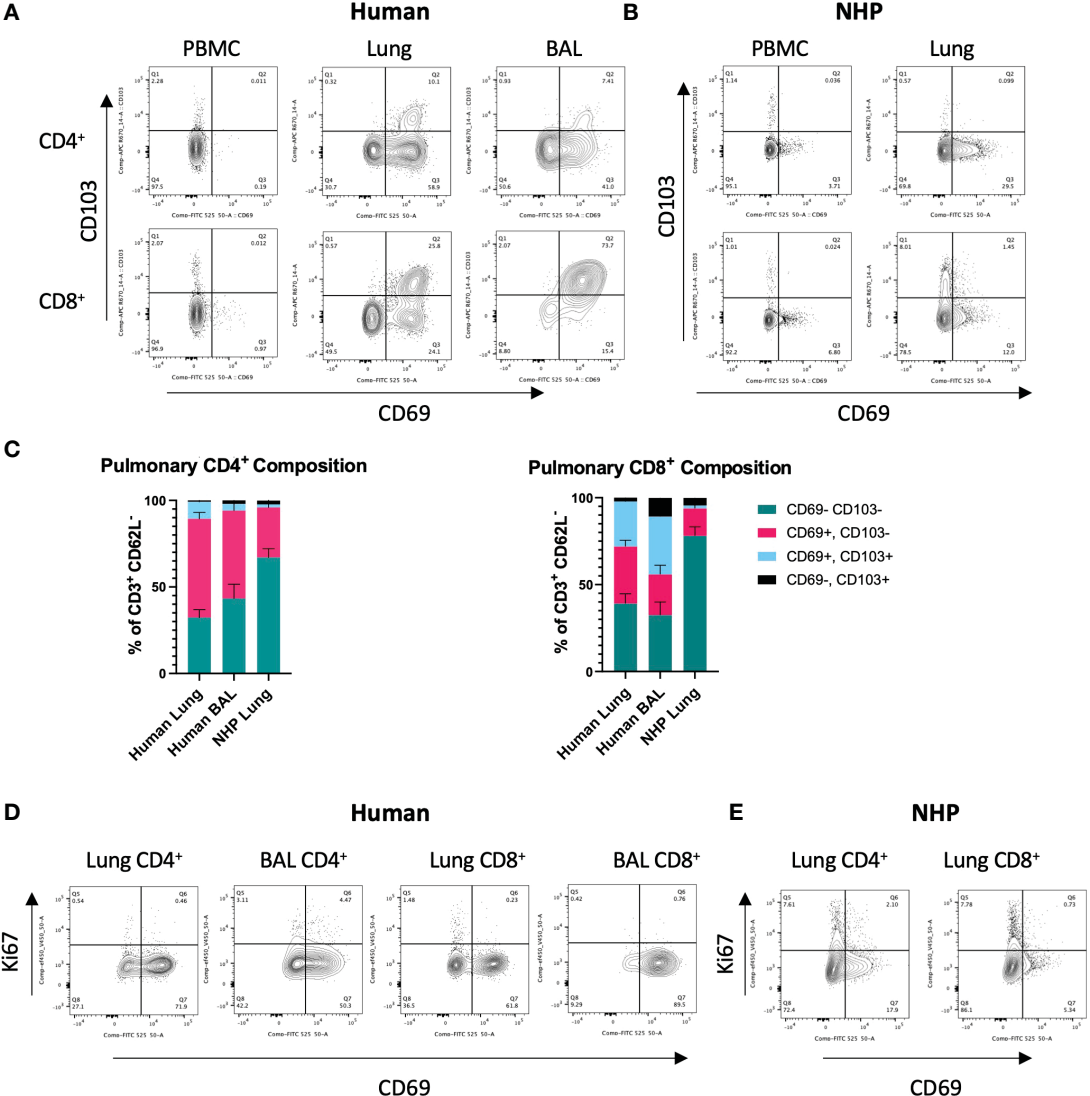
*Characterisation of Human and NHP Pulmonary T_RM._* T_RM_ from BAL and/or tissue digests were compared with peripheral T cells. **(A)** Identification of human pulmonary CD4^+^ and CD8^+^ T_RM_. T_RM_ (live, CD3^+^, CD4^+^/CD8^+^, CD62-L^-^) were identified based on their expression of CD69 and CD103. **(B)** Identification of NHP CD4^+^ and CD8^+^ T_RM_. **(C)** Proportion of CD69^+^ and CD69^+^CD103^+^ T_RM_ in human and NHP lung. **(D)** Ki-67 staining in human CD4^+^ and CD8^+^ T_RM_. **(E)** Ki-67 staining in NHP CD4^+^ and CD8^+^ T_RM_. Human and NHP samples were concatenated for analysis. Data represented as representative flow cytometry plots or compiled frequencies with mean ± SEM **(C)** from human lungs (n=15), BAL (n=9) and NHP lung (n=5).

Using a bronchoscope, the Panoptes multifunctional fibre was navigated to the distal airways *via* autofluorescence intensity imaging to visualise airways and alveoli (**Figure 1E**). 5x10^6^ pre-labelled human T_RM_ or B_RM_ were prepared as outlined in the previous section. For *in situ* T_RM_/B_RM_ detection, 20 µL (2 µg) CD69 + 20 µL (0.5 µg) CD103 or 20 µL (0.8 µg) CD20 + CD69 was diluted in 500 µL PBS. Antibodies or pre-labelled cells were instilled into different alveolar segments (to prevent cross contamination) of human lungs undergoing EVLV *via* the delivery channel of Panoptes Multifunctional Fibre. Fluorescence intensity and lifetime imaging was performed after 5 minutes using KronoScan OEM.

### Statistics

All data in text are represented as mean ± standard deviation (SD). All data in figures are represented as mean ± standard error of the mean (SEM). All graphs were generated using GraphPad Prism 9 (GraphPad Software, California, USA).

## Results

### Characterisation of human and NHP pulmonary T_RM_

To identify and characterise human and NHP pulmonary T_RM_, cells isolated from lung tissue were stained with cross-reactive antibodies for human and cynomolgus macaque and analysed by flow cytometry. Based on the existing knowledge (5), T_RM_ were phenotyped as CD3^+^, CD4^+^/CD8^+^, CD62-L^-^, CD69^+^, ± CD103 (for T_RM_ gating strategy, see **Supplementary Figure 2**). Exclusion of CD62-L^+^ (L-selectin) cells helped eliminate any contaminating circulating T_CM_ present within the vasculature of the lung tissue.

CD4^+^ and CD8^+^ T_RM_ were detected in human lung and BAL but were expectedly absent in peripheral blood (**Figure 1A**). The highest levels of CD103 expressing cells were seen in CD8^+^ T_RM_ in the BAL where CD69^+^CD103^+^ T_RM_ were the most abundant population (33.3% ± 22.9, **Figures 1A, C**). CD69^+^ CD4^+^ and CD8^+^ T_RM_ were also detected in NHP lung, although at much lower levels than that found in human lung (**Figures 1B, C**). 57% ± 14.3 of CD4^+^ T cells in human lung were positive for CD69 compared to 28% ± 11 in NHP lung. 32.8% ± 14 of CD8^+^ T cells in human were positive for CD69 compared to 15.8% ± 11.7 in NHP lung. There were some similarities in the expression of CD69 and CD103 in human and NHP samples: CD69^+^ T cells were present at higher frequencies in lung versus peripheral blood (**Figures 1A, B**) and lung CD8^+^ T cells showed higher levels of CD103 expression than their CD4^+^ counterparts in both species (**Figure 1C**). However, the overall frequency of CD69^+^ T_RM_ (± CD103) was lower in NHP than in human samples and co-expression of CD69 and CD103 was rarely observed in NHP (**Figures 1A–C**). Human lung contained 66.9% ± 17.6 and 58.7% ± 21.8 CD4^+^ and CD8^+^ T_RM,_ respectively, compared to 30.6% ± 11.9 and 17.4% ± 12.3 in NHP lung. In NHP lung, only 1.74% ± 1.9 of CD4^+^ and 1.52% ± 0.8 of CD8^+^ cells co-expressed CD69 and CD103, compared to 9.9% ± 10.7 CD4^+^ and 25.9% ± 17.1 CD8^+^ cells within human lung. CD62L^-^CD69^-^CD103^-^ T_EM_ were the largest population observed in NHP lung, with 67% ± 17.5 of CD4^+^ T cells and 78% ± 21.6 of CD8^+^ T cells failing to express tissue-residency markers.

To assess rates of proliferation, intracellular levels of Ki-67 were measured in pulmonary T_RM_. Low levels of Ki-67 were observed in human CD4^+^ and CD8^+^ T cells isolated from lung and BAL (**Figure 1D**). Ki-67 levels were slightly higher in NHP lung, particularly in CD69^-^ T cells, in line with their effector memory profile (**Figure 1E**).

Despite differences in expression of CD69 and CD103 between species, expression patterns of other markers associated with T_RM_ phenotype, CD49a and programmed cell death protein 1 (PD-1), were similar on human and NHP T_RM_. CD49a was absent on peripheral blood T cells but expressed by human and NHP CD69^+^ T_RM_, particularly those co-expressing CD103 (**Supplementary Figure 3**). Increased levels of PD-1 were also observed on human and NHP T_RM_, with expression highest on CD103^+^ T_RM_ and T_RM_ isolated from BAL (**Supplementary Figure 3**).

### Characterisation of human and NHP pulmonary B_RM_

**Figure 2:**
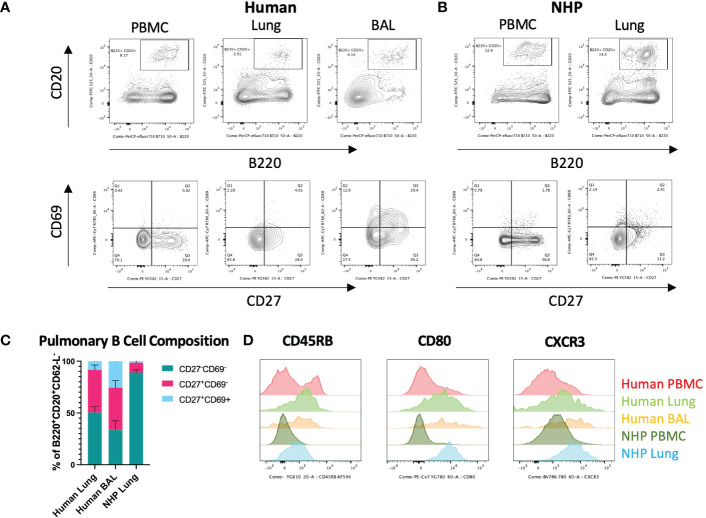
*Characterisation of Human and NHP Pulmonary B_RM_.* B_RM_ were isolated from BAL and/or tissue digests and compared with peripheral B cells. **(A)** Identification of human pulmonary B_RM_. B_RM_ (live, CD62L^-^, B220^+^, CD20^+^ (lower panels)) were identified based on their expression of CD27 and CD69. **(B)** Identification of NHP pulmonary B_RM_. **(C)** Proportion of B_RM_ in human and NHP lung. **(D)** Human and NHP B_RM_ surface marker expression. Human and NHP samples were concatenated for analysis. Surface marker expression was compared to B cells isolated from peripheral blood (PBMC). Data represented as representative flow cytometry plots, compiled frequencies with mean ± SEM or histograms from human lungs (n=9), BAL (n=7) and NHP lung (n=5).

Human and NHP lung cells were stained for pulmonary B_RM_ markers. B_RM_ were phenotyped as CD62-L^-^, B220^+^, CD20^+^, CD27^+^, CD69^+^ (5, 15) (for B_RM_ gating strategy, see **Supplementary Figure 4**). Both human and NHP lungs contained CD27^+^CD69^-^ memory B cells (B_MEM_) but a distinct population of CD27^+^CD69^+^ B_RM_ was mostly observed in human BAL (**Figures 2A, B**). CD27^+^ B_MEM_ were also detected in human and NHP peripheral blood, however these cells did not express CD69. Human lung and BAL contained similar frequencies of B_MEM_ (41.1% and 40.6%, respectively) (**Figure 2C**). NHP lung contained significantly fewer B_MEM_, with only 9.5% present. Human BAL contained the highest proportion of B_RM_, with 25.6% of memory B cells expressing CD69 compared to 8.4% in human lung and only 1.4% in NHP lung (**Figure 2C**). Although BAL contained the highest proportion of B_RM_, numbers were far fewer than those found in the lung interstitium.

Despite being fewer in frequency, NHP B cells from lung digests shared a similar surface marker phenotype to their human counterparts, expressing increased CD45RB, CD80 and CXCR3 levels compared to circulating B cells (**Figure 2D**).

### T_RM_/B_RM_ tissue immunofluorescence

Having identified the most robust markers and cross-reactive antibodies for detecting human and NHP T_RM_ and B_RM_, frozen lung sections were stained for immunofluorescence imaging. Acetone-fixed frozen sections were used due to the sensitivities of CD69 and CD103 epitopes to formalin fixation. Tyramide signal amplification was also used as all primary antibodies were raised in mouse. Therefore, we investigated the localization of CD103^+^ and CD69^+^ CD4^+^ and CD8^+^ T_RM_ and CD20^+^CD27^+^CD69^+^ B_RM_ in human and NHP lungs.

**Figure 3:**
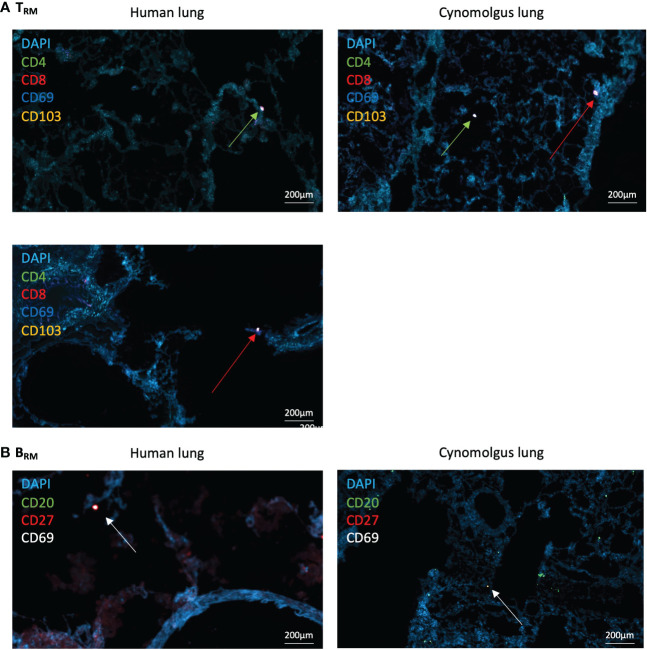
*Human and NHP Immunofluorescence T_RM_/B_RM_ Detection.* Frozen lung sections or BAL cells were stained using tyramide signal amplification. **(A)** Human and NHP T_RM_ detection in lung. CD4^+^ and CD8^+^ T_RM_ were detected based on their expression of CD69 and CD103. **(B)** Human and NHP B_RM_ detection in lung. B_RM_ were detected based on their expression of CD20, CD27 and CD69. Images taken at x400 magnification. Green arrows represent CD4^+^ T_RM_ and red arrows represent CD8^+^ T_RM_ **(A)**, and white arrows represent B_RM_ **(B)**.

CD69^+^ and CD69^+^CD103^+^ CD4^+^ and CD8^+^ T_RM_ were identified within both human and NHP distal airway (**Figure 3A** – for individual fluorescent channels, see **Supplementary Figures 5**, **6**). CD69^-^CD103^-^ CD4^+^ and CD8^+^ T cells were also evident. CD20^+^CD27^+^CD69^+^ B_RM_ were located within human and NHP distal lung, with less observed in NHP lung, mirroring flow cytometric results (**Figure 3B**). High frequencies of CD20^+^CD27^-^CD69^-^ B cells were also observed in NHP lung, again mirroring flow cytometric results. No evidence of repair-associated memory depots (RAMD) or inducible bronchus-associated lymphoid tissue (iBALT) was apparent as shown by a lack of clusters of CD4^+^/CD8^+^ T_RM_ or CD20^+^ B cells observed by immunofluorescence. DAPI staining following both staining protocols appeared diffuse, suggesting deterioration of the acetone-fixed, frozen tissue from the numerous wash and heat steps.

### Fluorescence intensity and lifetime imaging of human pulmonary T_RM_/B_RM_

**Figure 4:**
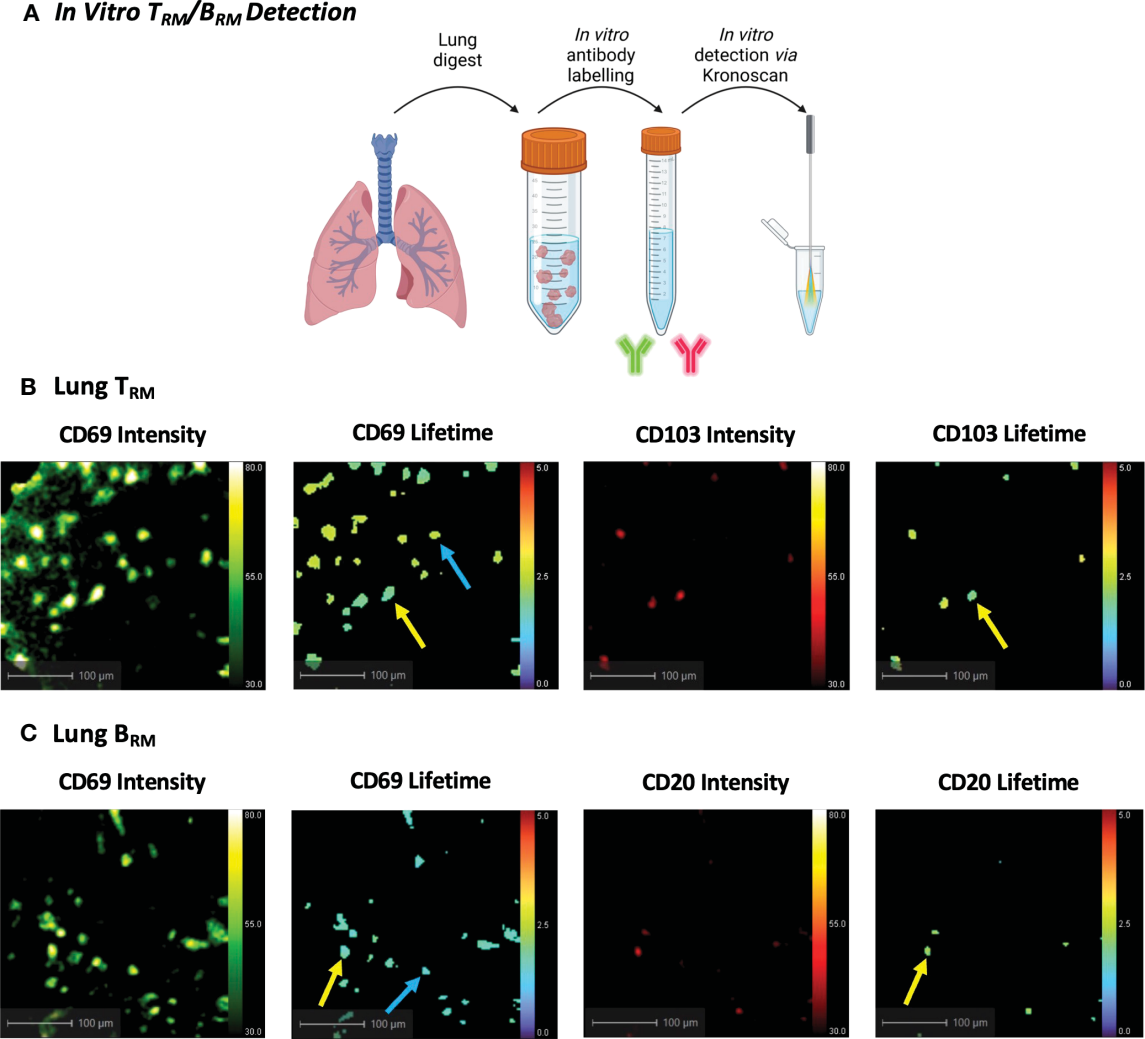
*In Vitro Human T_RM_ and B_RM_ Detection using KronoScan and Panoptes*. **(A)** Cells from human lung tissue digests were stained with T_RM_ or B_RM_ antibodies and imaged *in vitro* using KronoScan imaging system and Panoptes multifunctional fibre. **(B)** Human T_RM_ detection. T_RM_ were detected *via* expression of CD69 and CD103. Blue arrow indicates CD69^+^ cells and yellow arrow points CD69^+^CD103^+^ T_RM._ **(C)** Human B_RM_ detection. Blue arrow indicates CD69^+^ cells and yellow arrow points CD69^+^ CD20^+^ B_RM_. Representative images from n=3 experiments.

As the current KronoScan system is optimised for two excitation channels, we were unable to employ the lineage-based gating strategies commonly used in flow cytometry and could not definitively label T_RM_ in initial experiments. However, we assessed the potential of KronoScan to detect expression of CD69 and CD103, or CD69 and CD20, as the phenotypically distinguishing surface markers of T_RM_ and B_RM_, respectively. Utilising the excitation wavelengths (490 nm and 590 nm) and two spectral windows (498-565 nm, “green”, and 620-764 nm, “red”) of KronoScan, FITC-conjugated CD69 and PE-Dazzle conjugated CD103 or CD20 were selected for fluorescence intensity and lifetime imaging. As a proof-of-concept experiment, human lung digest cells were stained with fluorescent antibodies for CD69 and CD103 or CD20, and subsequently imaged *in vitro* with KronoScan (**Figure 4A**). Human pulmonary CD69^+^ and CD69^+^CD103^+^ cells could be visualised *via* both fluorescence intensity and lifetime imaging (**Figure 4B** + **Supplementary Video 1**) as well as CD69^+^CD20^+^ cells (**Figure 4C** + **Supplementary Video 2**). No fluorescence intensity or lifetime signal was observed from unstained lung cells in either the green or red channels (**Supplementary Figure 7A**). As expected, no CD69 or CD103 signal was detected from PBMCs (as negative controls) (**Supplementary Figure 7B** + **Supplementary Video 3**), however CD20^+^ B cells were observed (**Supplementary Figure 7C** + **Supplementary Video 4**).

### *In situ* detection of human pulmonary T_RM_/B_RM_

**Figure 5:**
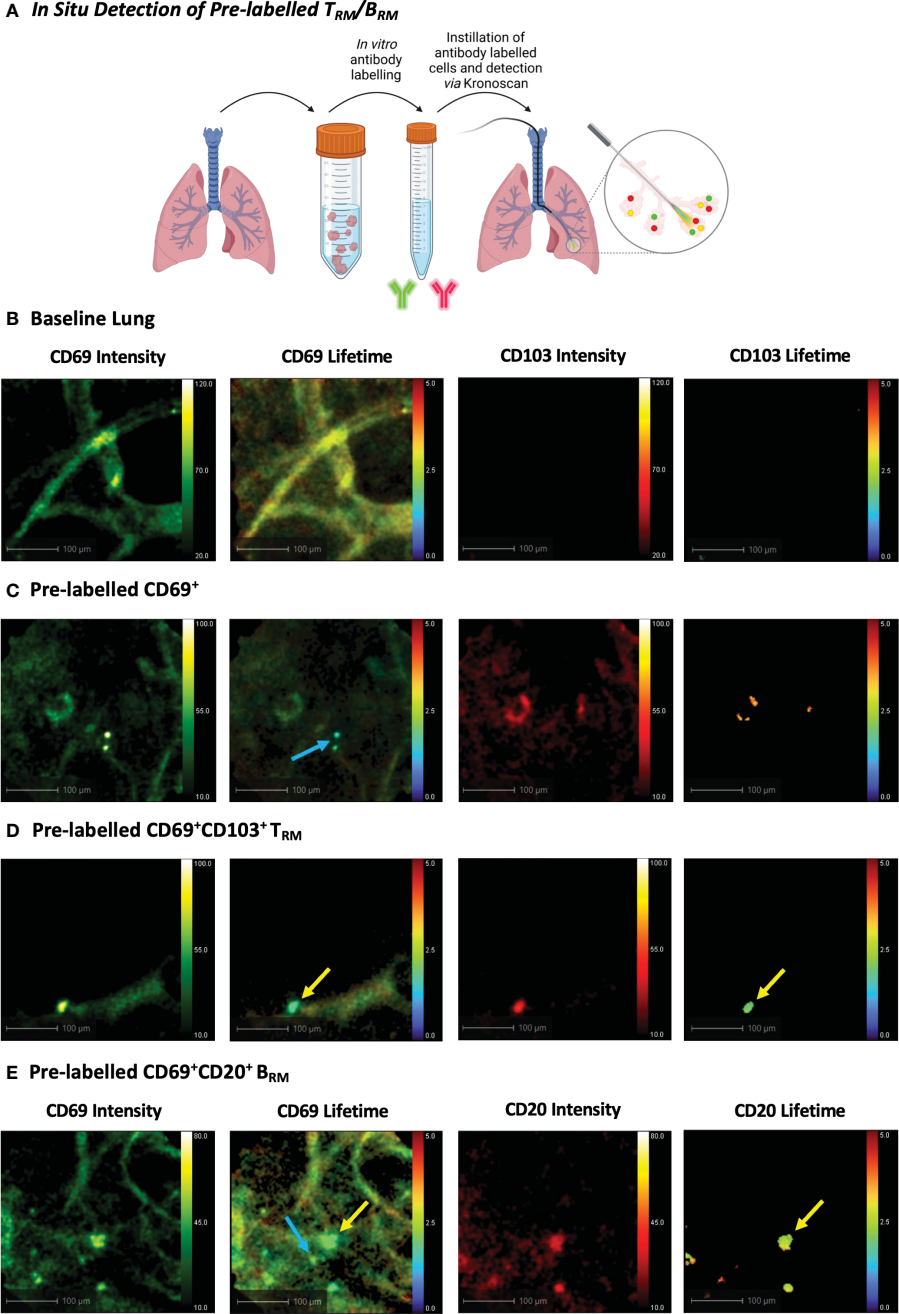
*In Situ Detection of Pre-labelled T_RM_/B_RM_ in Human Lung*. **(A)** Pre-labelled human pulmonary T_RM_ and B_RM_ in suspension were instilled into the alveolar space and detected using KronoScan and Panoptes during human EVLV. **(B)** Baseline lung imaging prior to pre-labelled cell instillation. **(C)** *In situ* imaging of pre-labelled CD69^+^ cells. Lung tissue digest cells were stained with fluorescent CD69 and CD103 antibodies prior to instillation into the alveolar space using Panoptes. Cells were imaged with KronoScan. Blue arrow indicates CD69^+^ cells. **(D)** *In situ* imaging of pre-labelled CD69^+^CD103^+^ T_RM_. Yellow arrow indicates CD69^+^CD103^+^ T_RM_. **(E)** *In situ* imaging of pre-labelled B_RM_. Lung tissue digest cells were stained with fluorescent CD20 and CD69 antibodies prior to instillation to the alveolar space. Blue arrow indicates CD69^+^ cells and yellow arrow points CD69^+^CD20^+^ B_RM_. Representative images from n=3 experiments.

To observe whether KronoScan and Panoptes could detect pulmonary CD69^+^/CD103^+^ and CD69^+^/CD20^+^ cells *in situ*, single cell suspensions from human lung digest were pre-stained for CD69 and CD103 or CD69 and CD20 and instilled into the alveolar space of lungs undergoing EVLV (**Figure 5A**). Fluorescence intensity imaging with 490 nm excitation was used to confirm that the Panoptes multifunctional fibre was within the alveolar space prior to instillation (**Figure 5B** + **Supplementary Video 5**). Alveolar lung architecture could be visualised *via* elastin autofluorescence of alveolar septae. No fluorescence intensity or lifetime signal was detected from lung tissue in the red channel. Upon instillation of labelled cells, CD69^+^ (**Figure 5C** + **Supplementary Video 6**), CD69^+^CD103^+^ cells (**Figure 5D** + **Supplementary Video 7**) and CD69^+^CD20^+^ cells (**Figure 5E** + **Supplementary Video 8**) could be visualised *via* an increase in fluorescence intensity counts as well as a lower lifetime signature of labelled cells that differentiated them from alveolar tissue and intrinsic unlabelled tissue cells.

**Figure 6:**
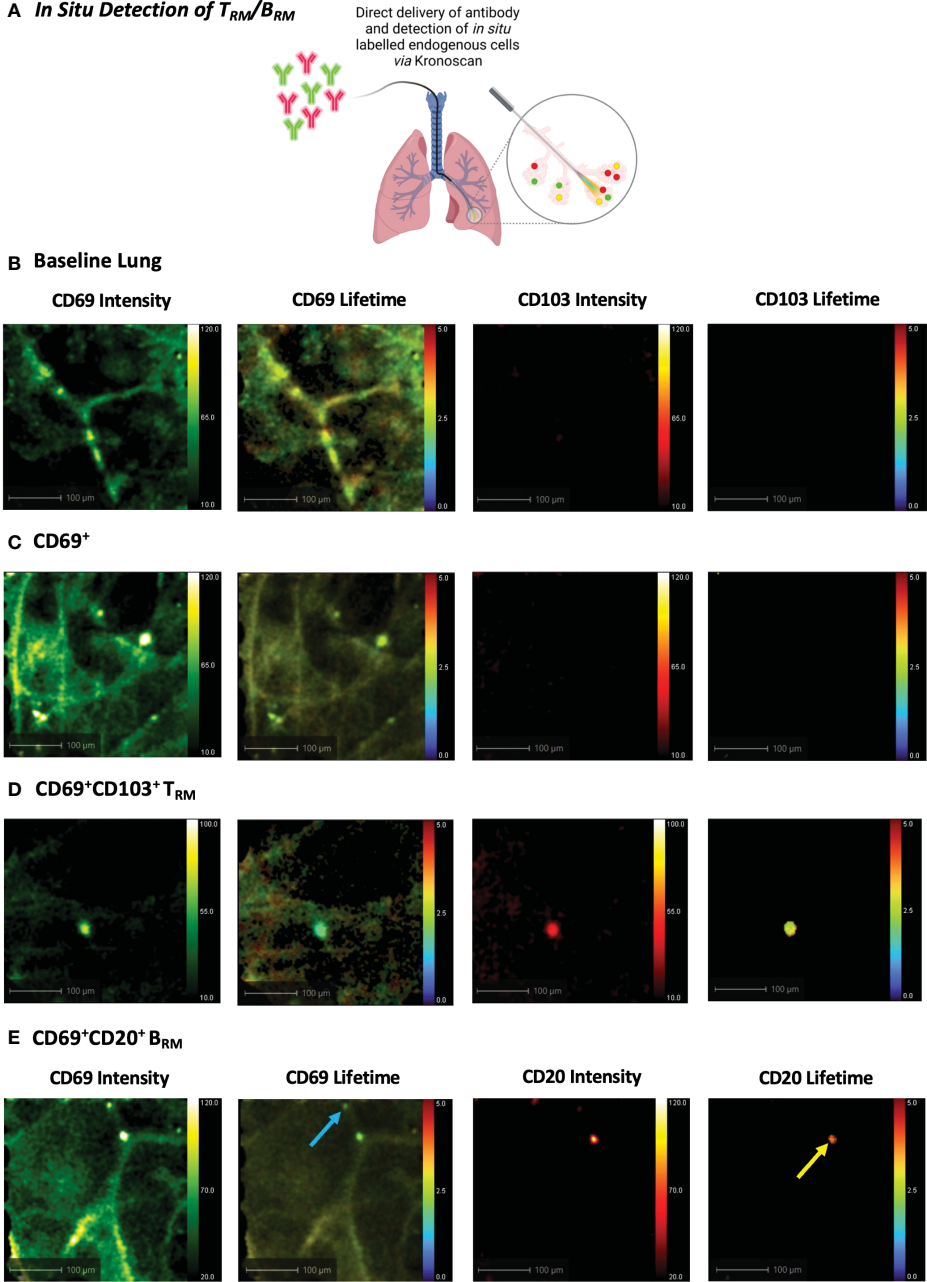
*In Situ Detection of T_RM_/B_RM_ in Human Lung following Antibody Delivery.* **(A)** T_RM_ or B_RM_ antibodies were instilled into the alveolar space *via* Panoptes fibre and imaged with KronoScan. **(B)** Baseline lung imaging prior to antibody delivery. **(C)** *In situ* detection of CD69^+^ cells. CD69 and CD103 antibodies were instilled into the alveolar space to detect T_RM_. **(D)** *In situ* detection of CD69^+^CD103^+^ T_RM_. **(E)** *In situ* detection of B_RM_. CD20 and CD69 antibodies were instilled into the alveolar space to detect B_RM_. Blue arrow indicates CD69^+^ cells and yellow arrow points CD69^+^CD20^+^ B_RM_. Representative images from n=3 experiments.

We next progressed to *in situ* labelling and detection of CD69^+^CD103^+^ and CD69^+^CD20^+^ cells following *intra-alveolar* delivery of microdoses (0.5–2 µg) of fluorescently labelled antibodies (**Figure 6A**). Baseline lung imaging confirmed that Panoptes was successfully navigated to the distal lung (**Figure 6B**). An instantaneous and rapid rise in fluorescence intensity counts confirmed successful delivery of the fluorescent antibody solution within the alveolar space (**Supplementary Video 9**). Within 120 seconds of CD69 and CD103 antibody delivery, CD69^+^ cells and CD69^+^CD103^+^ cells were visible *via* increased fluorescence intensity counts and a lifetime signature lower than that of lung tissue (**Figures 6C, D** + **Supplementary Videos 10**, **11**). Pulmonary B_RM_ were also visualised within the airways and alveolar space following instillation of CD69 and CD20 antibodies (**Figure 6E** + **Supplementary Video 12**).

## Discussion

Using cross-reactive antibodies for CD69 and CD103, we identified pulmonary T_RM_ in the lungs of humans and cynomolgus macaques. Both cell surface markers display key properties in the lung. CD69, although described as a marker of recent T cell activation induced through antigen stimulation and inflammatory cytokine exposure, supports effector T cell retention *via* the downregulation of sphingosine-1-phophsate receptor (S1PR1) and is considered as the main residency marker of pulmonary T_RM_ (30, 31). CD103, preferentially expressed on pulmonary CD8^+^ T_RM_, promotes adherence to E-cadherin and contributes towards initial recruitment and persistence to aid surveillance (32). Moreover, human and NHP CD4^+^ and CD8^+^ CD69^+^ T_RM_ expressed CD49a, particularly those co-expressing CD103. This integrin, specific for collagen IV, may contribute to the functionality of T_RM_ in the lung by facilitating locomotion for surveillance and through its critical role in cell survival by limiting apoptosis (32). Increased levels of PD-1 were also detected on human and NHP T_RM_, particularly on CD8^+^ T_RM_ and those isolated from human BAL. This marker may serve as a protective mechanism to prevent aberrant activation and excessive inflammation (33).

Although CD69^+^ T cells were identified in NHP lung with a higher frequency than in PBMC, there were notably fewer T_RM_ than were found in human lung or BAL. High levels of pulmonary CD69^+^ T_RM_ have been observed by others in Rhesus macaque, however CD69^+^CD103^+^ T_RM_ frequencies were low, similar to our study, even post vaccination (34). We obtained lungs from healthy control animals bred in clean laboratory conditions, which differ greatly to human antigen experienced lungs. Human T_RM_ frequencies are consistently higher than in mice maintained in clean conditions (35). In fact, CD69 expression in “dirty” pet store mice is more like that of humans (36). Limited pathogen exposure may therefore account for the reduced frequencies seen in this study. Moreover, T_RM_ have been described to undergo “retrograde migration” to the mediastinal lymph node where they provide longer-lived regional memory (37).This, combined with limited pathogen exposure, may contribute to a reduction in number of pulmonary T_RM_ in NHP macaques.

CD69 was also used to identify B_RM_ in the lungs of humans and NHP, which has been shown to be a marker of residency rather than recent activation (15, 38). However, expression levels were very low and CD69^+^ B_RM_ were only readily detectable in human BAL. Human and NHP lung B cells shared a similar phenotype to those reported in mice, with expression of CD80 and the chemokine receptor CXCR3 (17), suggesting their ability to rapidly differentiate into antibody secreting plasma cells (39). CD45RB, expressed by human B_RM_ in the gut and tonsil, was also detected in our study on pulmonary B cells (40).

Human lung T_RM_ weakly expressed Ki-67, indicating low level proliferation. Expression was similar to that found in blood, mirroring previous results (41). Murine studies suggest interstitial CD8^+^ T_RM_ sustain airway T_RM_ through a process of homeostatic proliferation (42), however we observed increased proliferation in human BAL. Proliferation was higher in NHP lung T_RM_. Of note, one human sample obtained from an individual who had suffered cardiac arrest out of hospital and aspirated expressed high levels of Ki-67 in lung and BAL T_RM_ which may reflect significant lung injury and infection.

Pulmonary CD69^+^ CD103^+^ and CD69^+^ CD20^+^ human cells were successfully visualised *in situ via* fluorescence intensity and lifetime imaging using KronoScan and Panoptes OEM. The lung is highly autofluorescent in the green channel due to the presence of collagen and elastin (43) which can interfere with fluorescent signatures and mask positive signals when using fluorescence intensity imaging alone. In contrast to fluorescence intensity imaging, FLIM is insensitive to the concentration of probe bound to target, meaning only microdoses of antibody are required to visualise a target making FLIM particularly advantageous for lung endomicroscopy. Using both in combination allows for the imaging fibre to be successfully navigated to the alveolar space using fluorescence intensity imaging and FLIM used for the detection of labelled cells. This also allows for the use of commonly employed green channel fluorophores which may not be easily detected against background lung autofluorescence using fluorescence intensity imaging alone.

The direct instillation of fluorescently-tagged monoclonal antibodies into the lungs of living humans may raise ethical concerns. It will therefore be necessary to consider the functional properties of the antibodies and whether target engagement would directly affect ongoing local or systemic immune responses. Labelled humanised antibodies would be advantageous, whilst fluorescently labelled Fab or Fv antibody fragments lacking the Fc portion would prevent issues associated with antibody-dependent cellular cytotoxicity. In addition, as FLIM is largely independent of concentration, only very small amounts of antibody would be required. In this work the amount of antibody used (0.5 – 2 µg per experiment) is likely to fall within the microdose range and lack systemic effects when compared to the repeated mg/kg doses often used in immuno-oncology and is therefore likely to be well tolerated.

The lifetime signature of the fluorophores used in this study were lower than that of the lung tissue. Fluorescence lifetime is environmentally sensitive (43), meaning the temperature, oxygen saturation/hypoxia and pH within the lung will affect lifetime values and thus absolute lifetime values may differ from tissue to tissue. Thus, FLIM was used as a contrast tool for visualising fluorophores and labelled cells against the background lung rather than as an absolute measure of specific FLT values. The combination of high fluorescence intensity with a FLT value lower than that of background lung distinctly shows the presence of labelled T_RM_/B_RM_. The lifetime values of antibody-labelled cells are consistently lower than that of background lung tissue and are consistent between experiments (i.e., prelabelled or *in situ* labelling of both T_RM_ and B_RM_).

FITC conjugated antibodies have the most spectral similarity to endogenous elastin and tissue autofluorescence, yet the FITC-CD69 was readily distinguishable with high specificity with KronoScan. The high concentration of local cell-specific labelling has been shown to diminish FLT as a function of antibody labelling concentration on the cell surface and is consistent with self-quenching effects expected at high densities of FITC molecules (44).

Due to limited fluorophore combinations on CD103 (clone 2G5) (and the spectral detection capabilities of KronoScan), we used the Ber-ACT8 CD103 clone. Although the Ber-ACT8 CD103 clone did not work in our cynomolgus model, it has been successfully used in Rhesus macaque models (34) and so has good translational potential. In the future, altering the band pass filters on KronoScan will enable the use of other fluorophore/cross reactive antibody combinations. Increasing the number of filter sets will also increase the number of fluorophore combinations that can be used simultaneously, allowing for more information to be collected (e.g., identifying and differentiating CD4^+^ or CD8^+^ T_RM_, or differentiating T_RM_ and B_RM_ subpopulations) and unmixed using both intensity and lifetime measurements.

In the wake of the COVID-19 pandemic, the realization that disease-relevant local immune responses in the lung are not reflected in the blood prompted a rallying call to immunologists to recognize that tissues, rather than blood, are where immune cells function (45). Understanding and investigating immune processes *in situ* is therefore essential. Successful *in situ* labelling of immune cells after direct delivery of small amounts (microdoses) of antibody by fluorescence intensity/lifetime imaging extends the methods available for molecular imaging and visualising cells in the distal lung following vaccination and infection. The large number of directly conjugated antibodies available for flow cytometry should make this technique readily adaptable to study the major immune cell types present in alveolar tissues. Direct antibody labelling could also be coupled with the use of activation based smart-probes or *in situ* imaging of pathogens in experimental settings to visualise infection-driven inflammatory interactions. A recent call for the development of intranasal vaccines capable of eliciting greater mucosal immunity may help to overcome the shortcomings of current COVID vaccines and prevent viral transmission (46). This highlights the potential benefits of increasing tissue-based immunity. Assessing local immunity with our *in situ* detection method could enhance the evaluation of such vaccines.

Fluorescent intensity-based OEM has shown proof of concept for real-time alveolar imaging, when combined with imaging agents for neutrophil activation (23), gram-negative (25) and gram-positive (47) bacteria. This is however the first report of alveolar fibre based FLIM imaging in the human lung, demonstrating that combining intensity and FLIM delivers contrast enhancement and multiplexing capability above and beyond existing OEM platforms. The multifunctional Panoptes fibre platform, which is designed as a single use disposable device, enables *in situ* delivery of microdoses of imaging agents and therapies.

We established a cross reactive antibody staining panel for assessing pulmonary T_RM_ and B_RM_ in human and cynomolgus macaque. Many of the antibodies used are cross reactive to other NHP species, including rhesus macaque, and so offer great translational research potential. This work reveals similarities and differences between human and NHP pulmonary resident memory lymphocyte subpopulations and demonstrates the potential of *in situ* pulmonary optical imaging for rapidly detecting immunologically diverse cells.

## Data availability statement

The original contributions presented in the study are included in the article/**Supplementary Material**. Further inquiries can be directed to the corresponding authors.

## Ethics statement

The studies involving human participants were reviewed and approved by London - Central Research Ethics Committee 16-LO-1883. Written informed consent was not provided because this study involves the use of human lungs that are unfit for transplant. Specialist Nurses for Organ Donation (SNODs) approach relatives of potential donors and obtain authorisation for the use of the donor’s organs and tissues for transplantation using the standard NHS Authorisation form. Using the same authorisation form, the SNODs also obtain authorisation for the use of the donor’s organs and tissues removed but subsequently found to be unsuitable for transplantation for other purposes (i.e. research studies, education, training). The use of lungs in this study has been approved by an independent ethics committee and NHSBT, the body responsible for organ donation and transplantation across the UK.

## Author contributions

RO’C, KF, MC-R, FB, KD, and VP conceptualised the project. DH, RO’C, and KD provided methods. DH, RO’C, HS, TQ, EG, and BM performed the experiments. GW and JS provided materials and technical expertise. DH and HS performed the data analysis. DH wrote the manuscript. DH, RO’C, MC-R, FB, KD, and VP edited the manuscript. All authors contributed to the article and approved the submitted version.

## Supplementary material

Supplementary Video 1
*In Vitro Fluorescence Imaging of Pulmonary T_RM_.* Cells from human lung tissue digests were stained with T_RM_ antibodies and imaged using KronoScan imaging system and Panoptes multifunctional fibre.

Supplementary Video 2
*In Vitro Fluorescence Imaging of Pulmonary B_RM_.* Cells from human lung tissue digests were stained with B_RM_ antibodies and imaged using KronoScan imaging system and Panoptes multifunctional fibre.

Supplementary Video 3
*Human PBMC CD69 CD103 In Vitro Fluorescence Imaging.* Human PBMCs were stained with T_RM_ antibodies and imaged using KronoScan imaging system and Panoptes multifunctional fibre.

Supplementary Video 4
*Human PBMC CD69 CD20 In Vitro Fluorescence Imaging.* Human PBMCs were stained with B_RM_ antibodies and imaged using KronoScan imaging system and Panoptes multifunctional fibre.

Supplementary Video 5
*Baseline Lung Fluorescence Imaging Prior to Pre-labelled Cell Instillation.* KronoScan and Panoptes imaging fibre was navigated to the alveolar space prior to pre-labelled cell instillation.

Supplementary Video 6
*In Situ Fluorescence Imaging of Pre-labelled Human Pulmonary CD69^+^ cells.* Lung tissue digest cells were stained with fluorescent CD69 and CD103 antibodies prior to instillation into the alveolar space using Panoptes. Cells were imaged with KronoScan.

Supplementary Video 7
*In Situ Fluorescence Imaging of Pre-labelled Human Pulmonary CD69^+^CD103^+^ T_RM_.* Lung tissue digest cells were stained with fluorescent CD69 and CD103 antibodies prior to instillation into the alveolar space using Panoptes. Cells were imaged with KronoScan.

Supplementary Video 8
*In Situ Fluorescence Imaging of Pre-labelled Human Pulmonary CD69^+^CD20^+^ B_RM_.* Lung tissue digest cells were stained with fluorescent CD69 and CD20 antibodies prior to instillation into the alveolar space using Panoptes. Cells were imaged with KronoScan.

Supplementary Video 9
*Baseline Lung Fluorescence Imaging Prior to Antibody Delivery.* KronoScan and Panoptes imaging fibre was navigated to the alveolar space prior to fluorescent antibody delivery.

Supplementary Video 10 In Situ Imaging of Human CD69^+^ Cells Following Antibody elivery. CD69 and CD103 antibodies were delivered to the alveolar space and subsequently imaged.

Supplementary Video 11 In Situ Imaging of Human CD69^+^CD103^+^ T_RM_ Following Antibody Delivery. CD69 and CD103 antibodies were delivered to the alveolar space and subsequently imaged.

Supplementary Video 12 In Situ Fluorescence Imaging of Human Pulmonary B_RM_ Following Antibody Delivery. CD69 and CD20 antibodies were delivered to the alveolar space and subsequently imaged.
