# Supplementary material for: Specific in situ immuno-imaging of pulmonary-resident memory lymphocytes in human lungs
Source: Front Immunol. 2023 Feb 10;14:1100161. doi: 10.3389/fimmu.2023.1100161 (PMC9951616; doi:10.3389/fimmu.2023.1100161)
Supplement: Supplementary file 1 [file DataSheet_1.pdf]

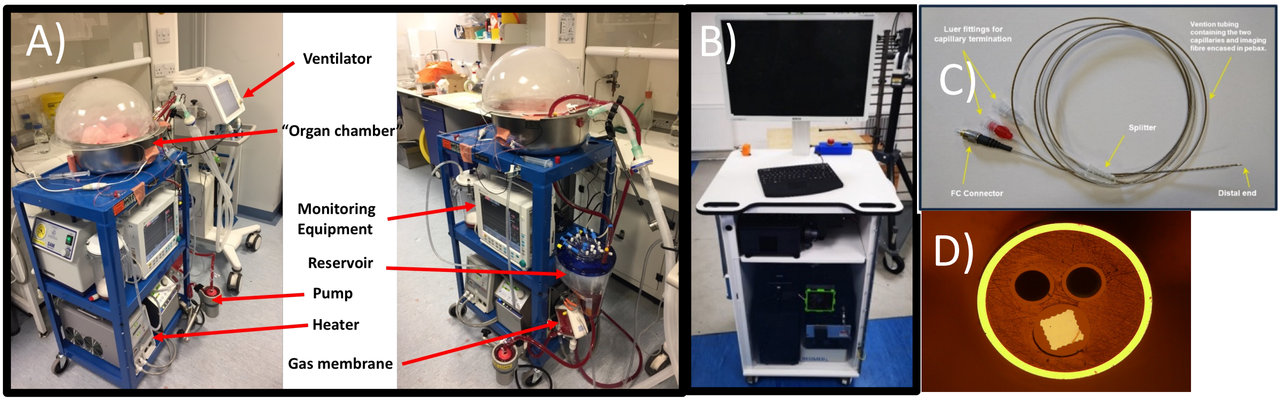


*Supplementary Figure 1 – Ex Vivo Lung Perfusion, KronoScan Imaging Device & Panoptes Imaging Fibre and in situ detection of pulmonary $T_{RM}$/$B_{RM}$.* A) *Ex Vivo Lung Ventilation (EVLV).* Human lungs were ventilated within the EVLV rig. B) KronoScan Imaging Device. C) Panoptes imaging fibre. Imaging fibre consists of 2 delivery/aspiration capillaries alongside imaging core containing over 8000 individual imaging fibres. Using a bronchoscope, the Panoptes imaging fibre can be navigated to the distal airways to deliver fluorescent imaging agents and visualise targets of interest. D) Magnified image of Panoptes imaging fibre.

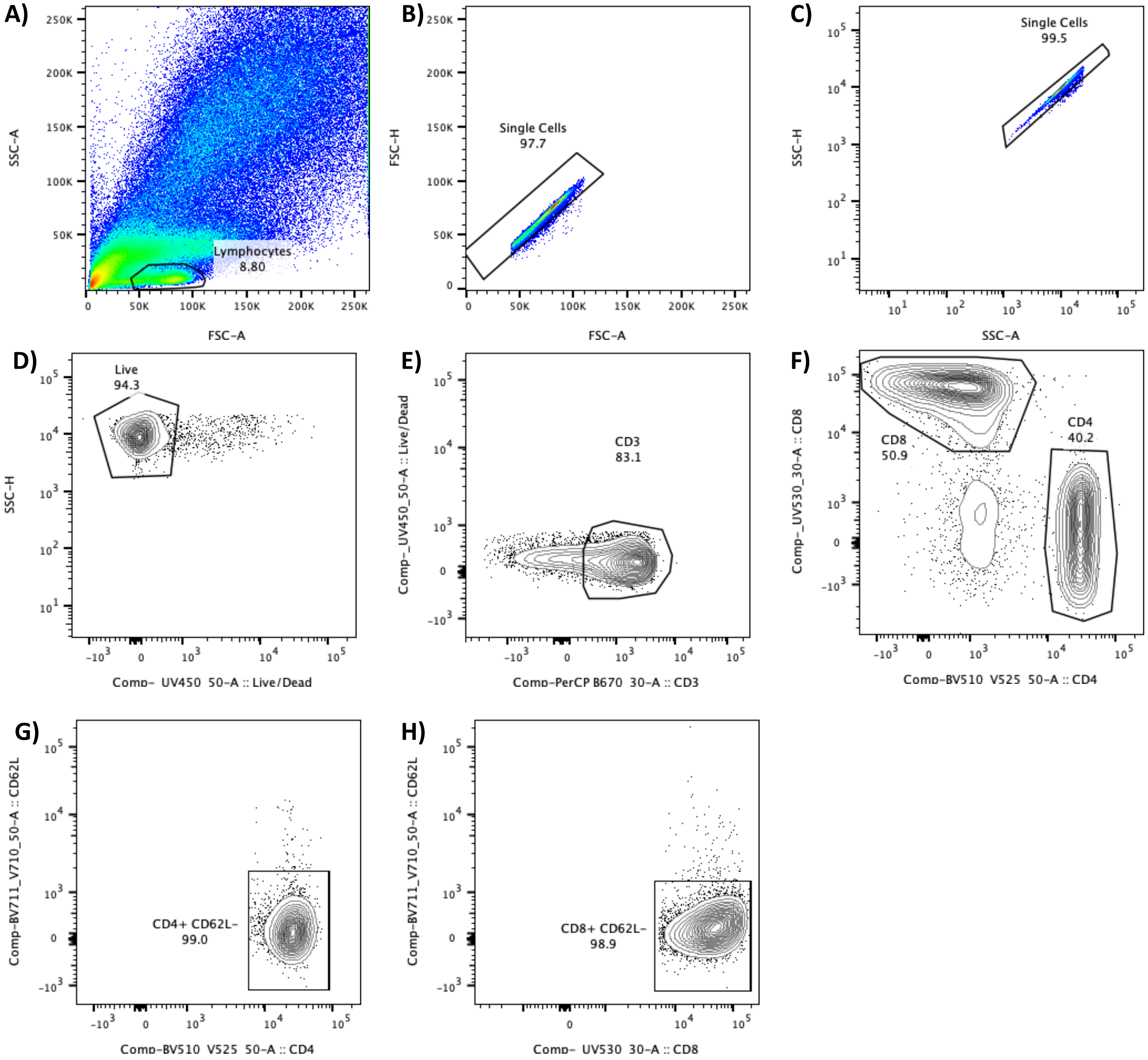


*Supplementary Figure 2 – Gating Strategy to Identify Pulmonary $T_{RM}$ (Lung + BAL).* Human and NHP $T_{RM}$ were gated based on their forward and side scatter profiles and considered single, live, $CD3^+$, $CD4^+$/$CD8^+$, CD62-$L^-$, $CD69^+$ ± CD103. Plots represented as representative flow cytometry plots.

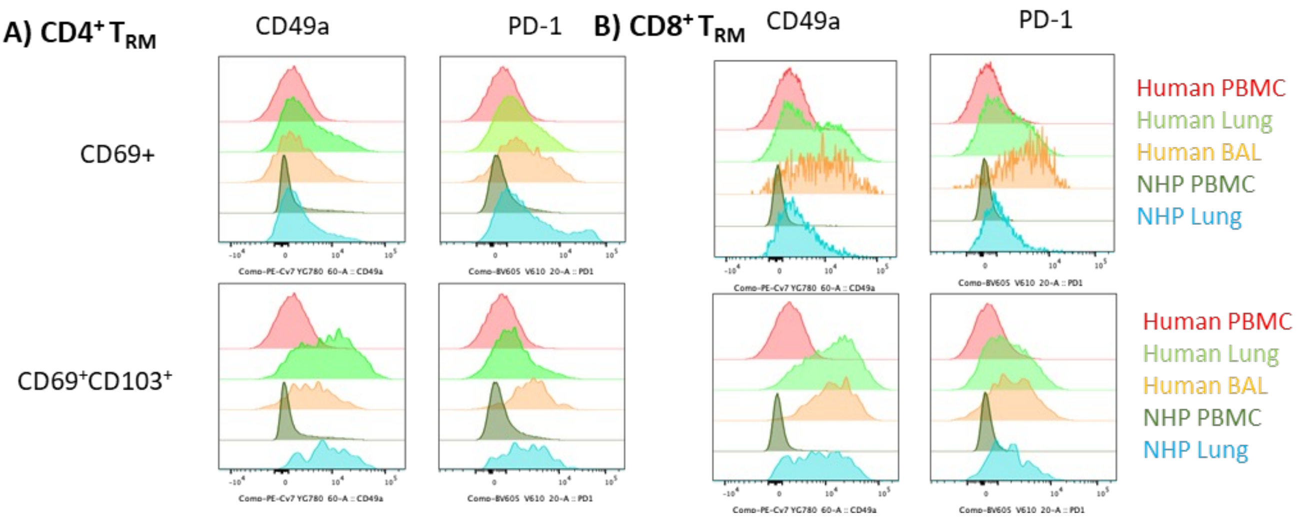


*Supplementary Figure 3 – Human and NHP CD4⁺ and CD8⁺ $T_{RM}$ surface marker expression.* Human and NHP samples were concatenated for analysis. Surface marker expression was compared with T cells isolated from peripheral blood (PBMC). A) CD4⁺ $T_{RM}$ surface marker expression. B) CD8⁺ $T_{RM}$ surface marker expression. Data represented as histograms from human lungs (n=9), BAL (n=7) and NHP lung (n=5).

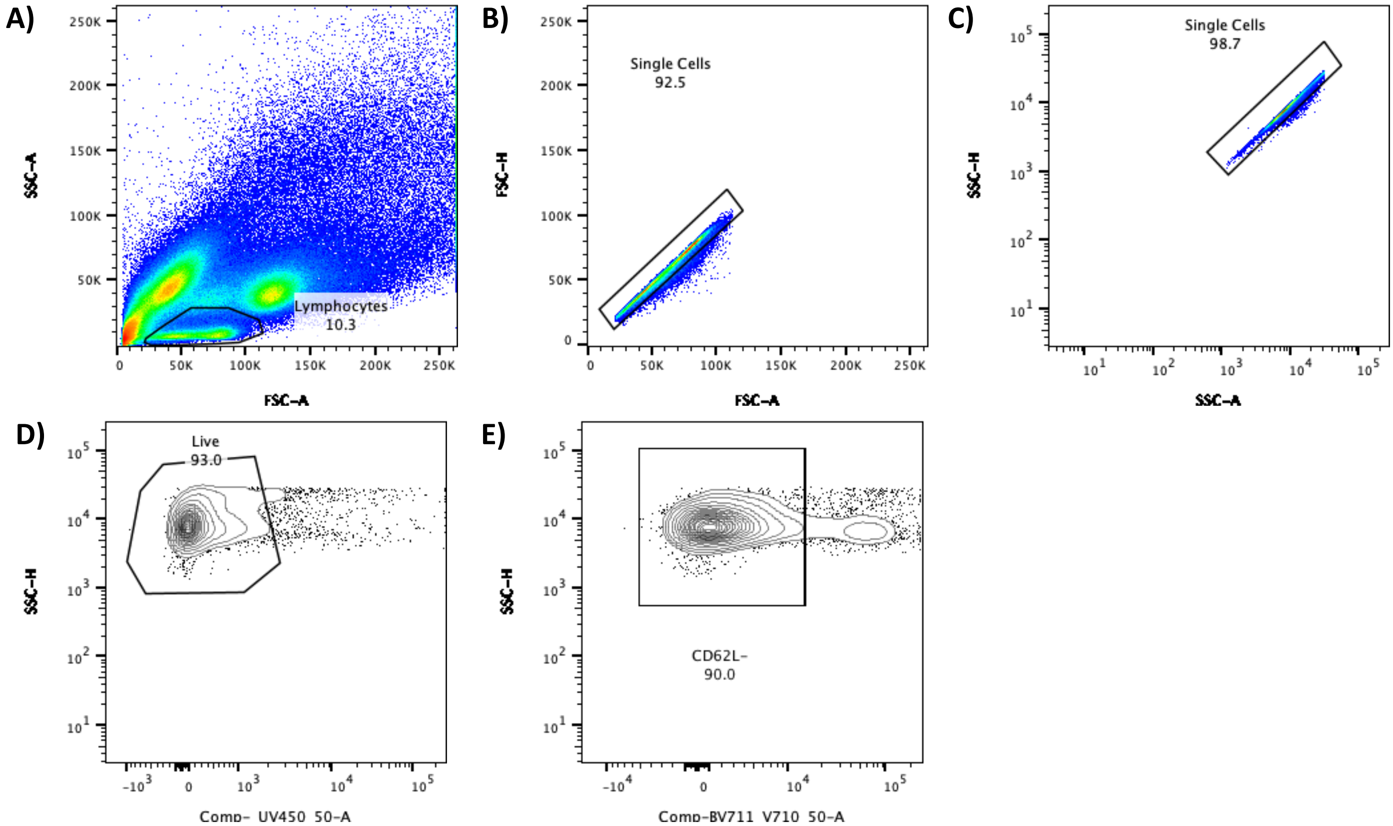


*Supplementary Figure 4 – Gating Strategy to Identify Pulmonary $B_{RM}$ (Lung + BAL).* Human and NHP $B_{RM}$ were gated based on their forward and side scatter profiles and considered single, live and CD62-L$^{-}$ , B220$^{+}$, CD20$^{+}$, CD27$^{+}$, CD69$^{+}$. Plots represented as representative flow cytometry plots.

A) Human CD4$^+$ T$_{RM}$

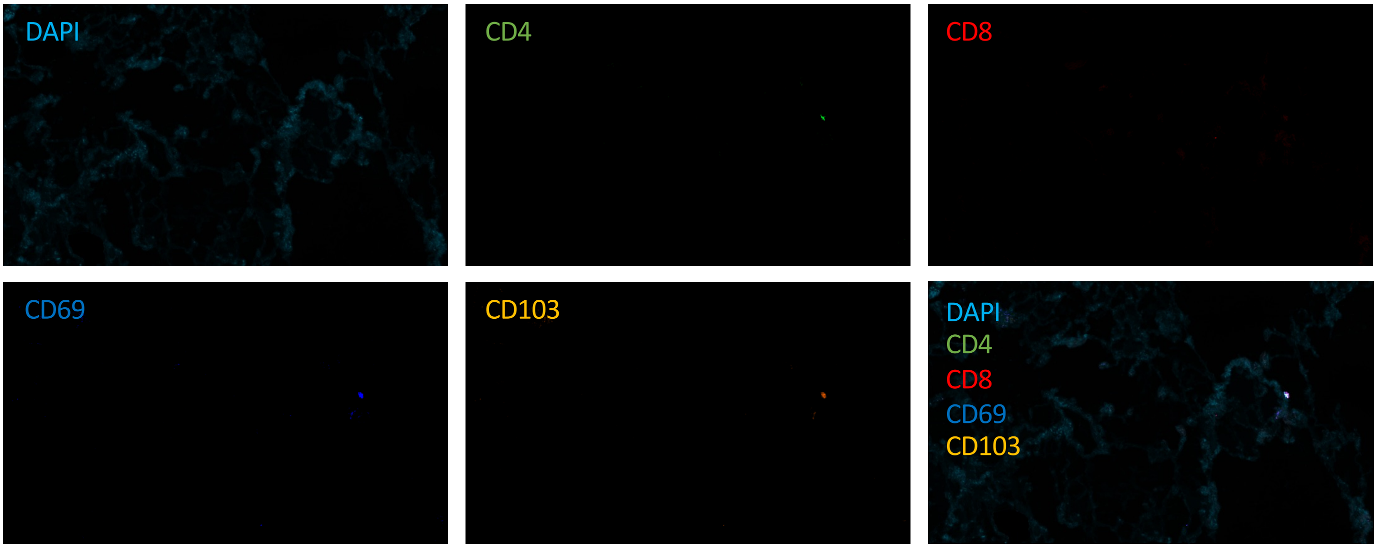


B) Human CD8$^+$ T$_{RM}$

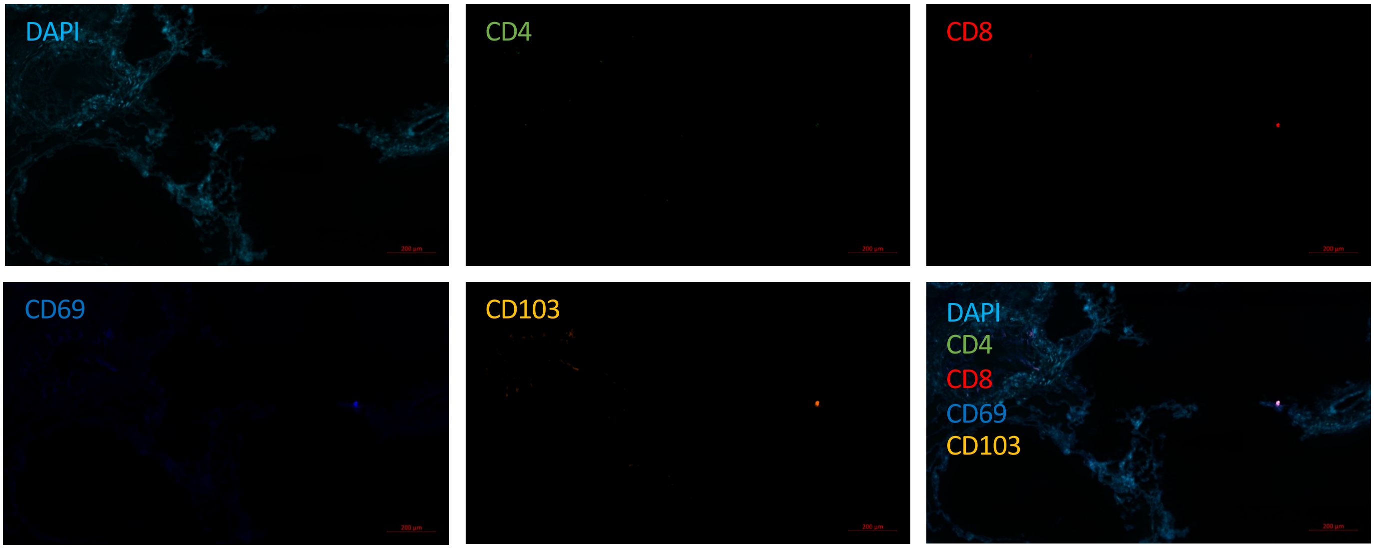


C) NHP CD4$^+$ + CD8$^+$ T$_{RM}$

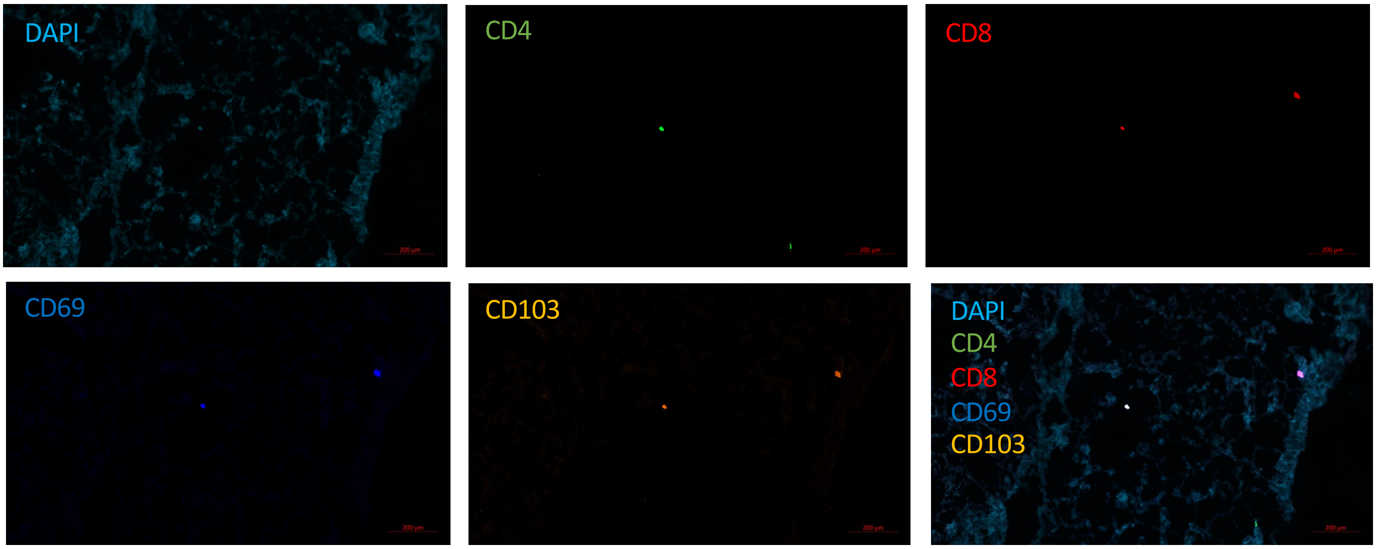


*Supplementary Figure 5– Individual T$_{RM}$ Immunofluorescent Fluorescent Channels.* Individual stains for DAPI, CD4, CD8, CD69 and CD103 alongside the merged image following fluorescence staining of human and NHP lung tissue. A) Human CD4$^+$ T$_{RM}$. B) Human CD8$^+$ T$_{RM}$. C) NHP CD4$^+$ + CD8$^+$ T$_{RM}$. Images taken at x400 magnification.

A) Human $B_{RM}$

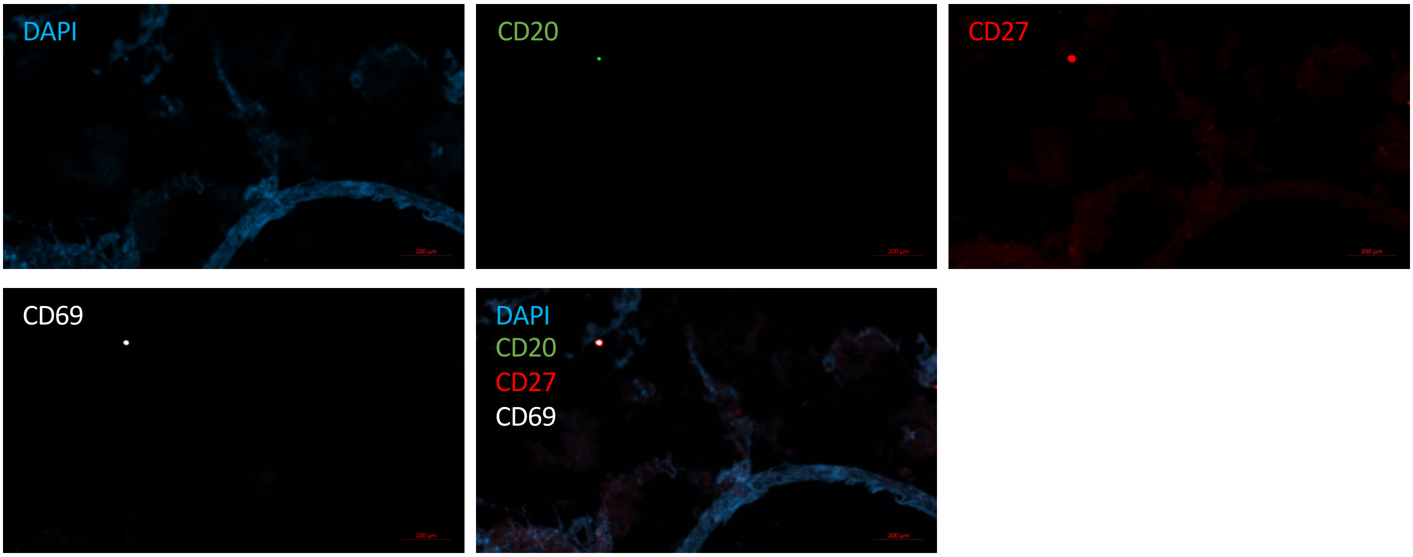


B) NHP $B_{RM}$

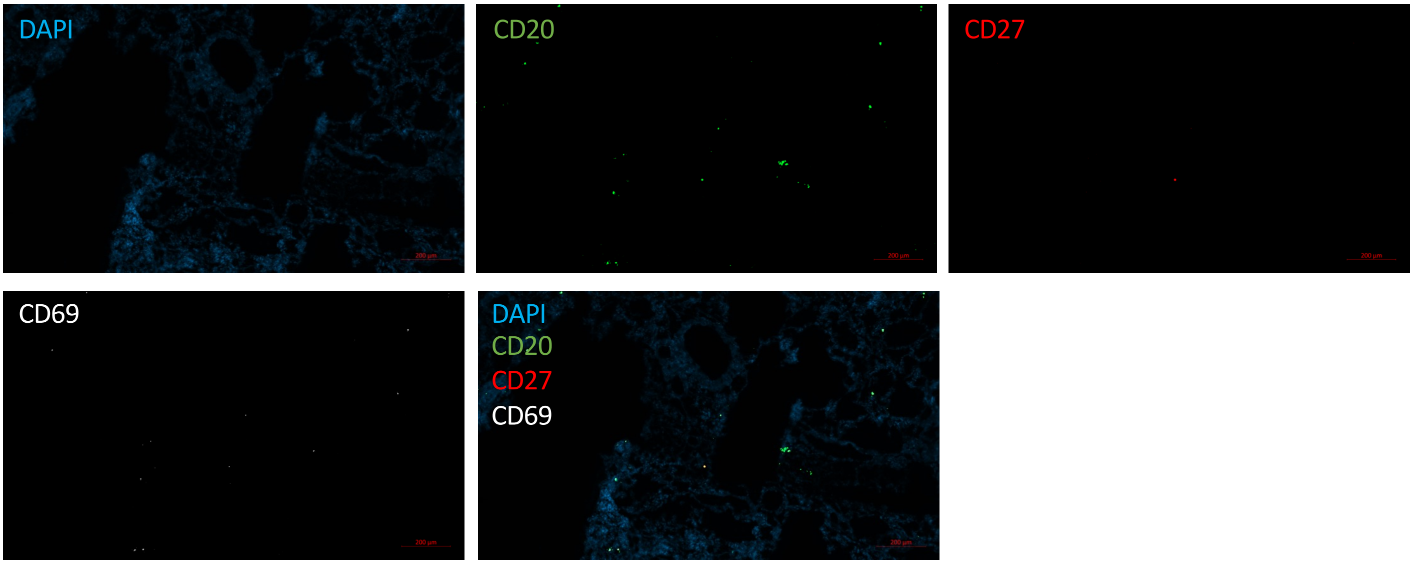


*Supplementary Figure 6 – Individual $B_{RM}$ Immunofluorescent Fluorescent Channels.* Individual stains for DAPI, CD20, CD27 and CD69 alongside the merged image following fluorescence staining of human and NHP lung tissue. A) Human $B_{RM}$. B) NHP $B_{RM}$. Images taken at x400 magnification.

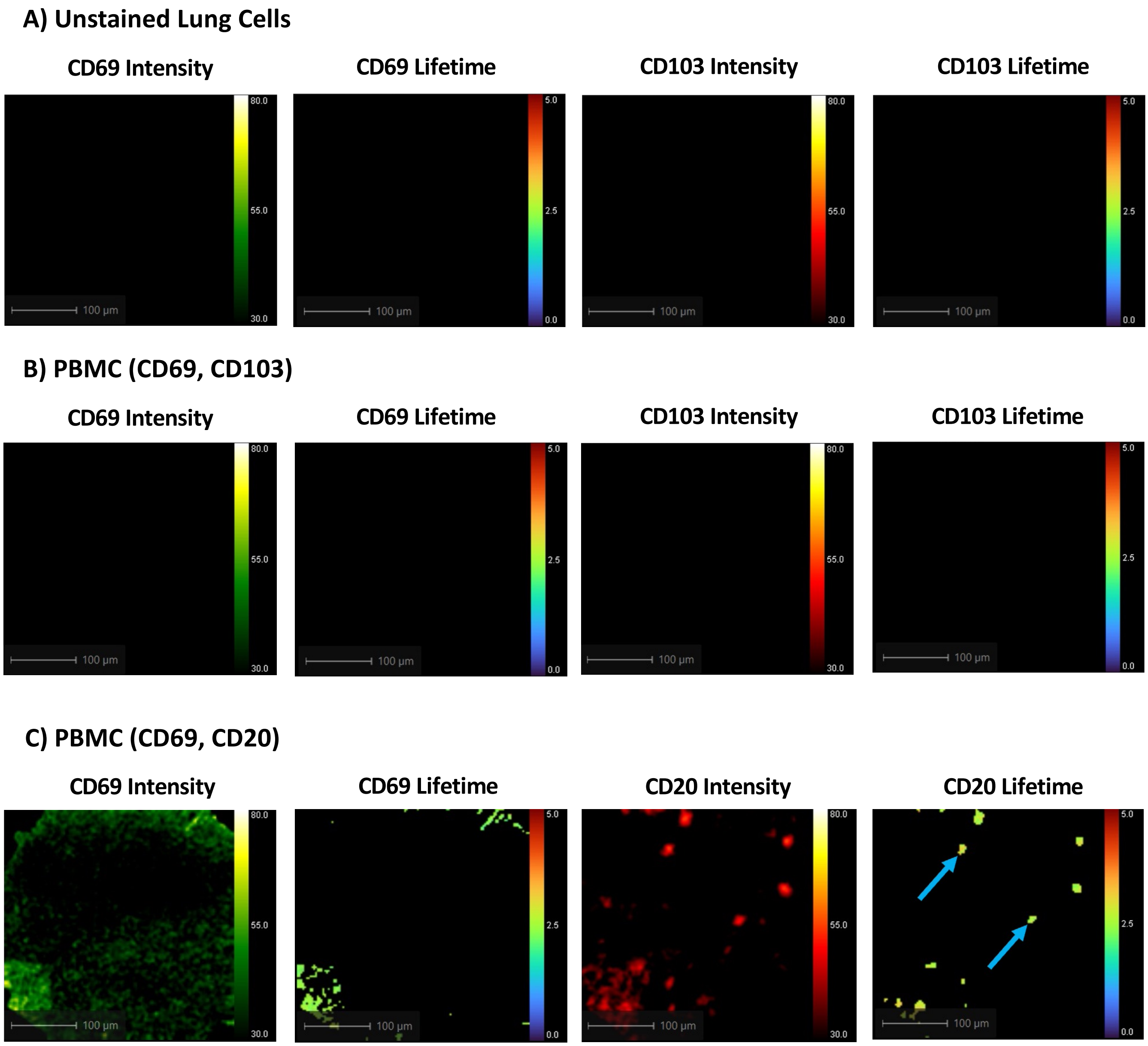


*Supplementary Figure 7 – Fluorescence Intensity and Lifetime Imaging of Human Lung Cells and PBMCs.* Human lung cells and PBMCs were stained for $T_{RM}$ or $B_{RM}$ and imaged using KronoScan and Panoptes. A) Fluorescence intensity and lifetime imaging of unstained lung cells. B) CD69 and CD103 staining of PBMCs. C) CD69 and CD20 staining of PBMCs. Blue arrows indicate the presence of $CD20^{+}$ B cells. Representative images.
