# Supplementary material for: Specific in situ immuno-imaging of pulmonary-resident memory lymphocytes in human lungs
Source: Front Immunol. 2023 Feb 10;14:1100161. doi: 10.3389/fimmu.2023.1100161 (PMC9951616; doi:10.3389/fimmu.2023.1100161)
Supplement: Supplementary file 2 [file DataSheet_2.pdf]

Online Data Supplement

Specific *In Situ* Immuno-imaging of Pulmonary-Resident Memory Lymphocytes in Human Lungs

Duncan Humphries, Richard O'Connor, Hazel Stewart, Tom Quinn, Erin Gaughan, Beth Mills, Gareth Williams, James Stone, Keith Finlayson, Martine Chabaud-Riou, Florence Boudet, Kevin Dhaliwal, Vincent Pavot.

T_{RM}/B_{RM} Immunofluorescence Staining: Frozen lung sections were inserted into the Bond-Rx Multiplex IHC Stainer (Leica). Sections were stained according to the following protocol (using 150µL reagent volumes at ambient temperature unless otherwise stated).

1. Universal protein block (30 minutes)
2. Universal peroxidase block
3. Universal peroxidase block (10 minutes)
4. Peroxide block
5. Primary A (CD4 or CD20) (30 minutes)
6. Universal secondary (30 minutes)
7. Tyramide 520 (10 minutes)
8. Heat wash pH9
9. Heat wash pH9
10. Heat wash (85°C, 20 minutes)
11. Universal protein block (30 minutes)
12. Universal peroxidase block

13. Universal peroxidase block (10 minutes)

14. Peroxide block

15. Primary B (CD8 or CD27) (30 minutes)

16. Universal secondary (30 minutes)

17. Tyramide 570 (10 minutes)

18. Heat wash pH9

19. Heat wash pH9

20. Heat wash (85°C, 20 minutes)

21. Universal protein block (30 minutes)

22. Universal peroxidase block

23. Universal peroxidase block (10 minutes)

24. Peroxide block

25. Primary C (CD69) (30 minutes)

26. Universal secondary (30 minutes)

27. Tyramide 620 (10 minutes)

28. Heat wash pH9

29. Heat wash pH9

30. Heat wash (85°C, 20 minutes)

31. For T_{RM} panel only - Universal protein block (30 minutes)

32. Universal peroxidase block

33. Universal peroxidase block (10 minutes)

34. Peroxide block

35. Primary D (CD103) (30 minutes)

36. Universal secondary (30 minutes)

37. Tyramide 690 (10 minutes)
38. Heat wash pH9
39. Heat wash pH9
40. Heat wash (85°C, 20 minutes)
41. DAPI counterstain
42. Following wash step, cells mounted using PermaFluor™ (Fisher Scientific)

Slides were scanned using an Axio Scan Z1 (Zeiss, Germany) or imaged using or a Axio Imager Z1 fluorescence microscope (Zeiss).
