# Supplementary material for: Specific in situ immuno-imaging of pulmonary-resident memory lymphocytes in human lungs
Source: Front Immunol. 2023 Feb 10;14:1100161. doi: 10.3389/fimmu.2023.1100161 (PMC9951616; doi:10.3389/fimmu.2023.1100161)
Supplement: Supplementary file 3 [file Table_1.docx]

| **Cell Type** | **Surface Marker** | **Antibody Clone** | **Fluorophore** | **Volume (µL)** | **Supplier** |
| --- | --- | --- | --- | --- | --- |
| **T_RM_** | CD3 | SP34-2 | PerCP | 20 | BD |
|  | CD4 | OKT4 | BV510 | 5 | Biolegend |
|  | CD8 | SK1 | BUV737 | 5 | BD |
|  | CD69 | FN50 | FITC | 5 | Biolegend |
|  | CD103 | 2G5 | APC | 5 | BC |
|  | CD49a | TS2/7 | PE-Cy7 | 5 | Biolegend |
|  | PD-1 | EH12.2H7 | BV605 | 5 | Biolegend |
|  | CD62-L | SK11 | BV711 | 5 | BD |
|  | Ki-67 | SolA15 | eFluor450 | 0.625 | Thermo |
|  |  |  |  |  |  |
| **B_RM_** | CD45RB | MEM-55 | AF594 | 5 | Biolegend |
|  | B220 | GRT22 | PerCP-eFlour710 | 5 | Thermo |
|  | CD20 | 2H7 | FITC | 5 | Biolegend |
|  | CD27 | M-T271 | PE | 5 | Biolegend |
|  | CD69 | FN50 | APC-Cy7 | 5 | Biolegend |
|  | **CD80** | **2D10** | **PE-Cy7** | **5** | **Biolegend** |
|  | CXCR3 | G025H7 | BV785 | 5 | Biolegend |
|  | CD62-L | SK11 | BV711 | 5 | BD |
|  | Ki-67 | SolA15 | AF700 | 1.25 | Thermo |

*Supplementary Table 1 – Cross-Reactive Antibody Panels.* ***Bold*** *= human-reactive only. BC = Beckman Coulter. BD = BD Biosciences. Thermo = Thermo Fisher Scientific*
